# Supplementary material for: Unique exploration method of electronic component failure time and yield strengths of circular tubes under complete flexible model
Source: PLoS One. 2024 Oct 14;19(10):e0310150. doi: 10.1371/journal.pone.0310150 (PMC11472960; doi:10.1371/journal.pone.0310150)
Supplement: Attachment — Submitted filename: Responses to Reviewers.pdf [file pone.0310150.s001.pdf]

Our Reply to Reviewers

Before we begin answering the comments of the two reviewers point by point, we would like to thank them for important comments we think they have been paved the way towards a significant improvement of the paper. Now, we will present our reply for specific comments.

- The referee comments are shown in black.
- Our responses for reviewer #1 are shown in red, and responses for reviewer #2 are shown in blue.
- Furthermore, the corrections in the revised version based on the comments of reviewer #1 are shown in red, whereas the corrections in the revised version based on the comments of reviewer #2 are shown in blue.

**Reviewer #1**	**Responses to Reviewer #1**
Major comments:	
The title of the paper is not according to the topic. I suggest the revision in the title	We modified the title of the article according to proposed work.
I suggest not to define the abbreviations in the abstract. Also once an abbreviation is defined should not be defined again or should not be used in full for the next time.	We excluded all abbreviation from abstract. We defined all abbreviations only one time.
The manuscript needs to a comprehensive improvement for its language and presentation.	Thanks for your comment. The English language and presentation of the paper are improved throughout the paper.
Why the author used new Kumaraswamy-Pareto (NKP) distribution as the baseline distribution of the EWMA chart.Give the motivation behind this.	As we can see this distribution is very flexible in its baseline article and can almost completely fitted on every data In that article it checked through goodness of fit measures that it is better fit as compare to various existing distributions Now anyone who wants to use our proposed charts in industry or agriculture sector, not

	only can applied our proposed control charts on any data with good results but also can show that this distribution is better fit, through goodness of fit measures, for this data.
Add some latest research articles in introduction.	We added latest articles in introduction.
Explain the motivation behind comparing LSE specifically with the WLSE.	The EWMA CC based on WLSE will be always better than EWMA CC based on LSE. There are two philosophies which run behind this argument. First philosophy is that WLSE is weighted form (modified form) of least square estimator. The second philosophy is that we have seen in its baseline article [1] that all estimators with all estimation methods, the WLSE is better than LSE.
Include some more graphical presentations of the results in the manuscript	We included half dozen plots and some 3D plots also.
Minor comments:	
The authors are suggested to Extend Table 1 by adding some more shifts.	We added shift value and obtained new ARLs values.
Check formatting specially besides Figures and Tables	We checked formatting specially besides Figures and Tables.
Figures should be interpreted in more details.	Figures interpreted in more details.
Conclusion is weak. It requires improvement.	Conclusion changed with improvement.
References should write with same style	References wrote with same style.

Reviewer #2	Responses to Reviewer #2
Define all acronyms at their first occurrence in the text.	We defined all acronyms at their first occurrence.
Provide more detailed explanations for the terms "least square estimator (LSE)" and "weighted least square estimator (WLSE)" in the introduction.	We provided detailed explanation for terms "least square estimator (LSE)" and "weighted least square estimator (WLSE)" in the introduction.
Clarify the significance of the shape parameter in the context of the NKP distribution	We clarified the significance of the shape parameter in the context of the NKP distribution
Include a brief overview of existing EWMA control charts to highlight the novelty of the proposed method.	We wrote a brief overview of existing EWMA control charts to highlight the novelty of the proposed method.
Add references to support the claims made about the flexibility of the NKP distribution	We added references to support the statements made about the NKP distribution.
Ensure that all figures are properly labeled and referenced in the text.	All figures are properly labeled and referenced in the text.
Explain the choice of parameters (α, γ, θ) used in the simulation study	We explained the choice of parameters (α, γ, θ) used in the simulation study.
Discuss the potential limitations of the proposed control charts.	We made separate heading of limitations of the proposed control charts.
Provide more context on the selection of the two real data sets used for validation.	We discussed two data sets in detail.
Elaborate on the implications of the results obtained from the simulation study.	The discussed results obtained from the simulation study in detail.
Include a table summarizing the key findings from the comparative analysis of EWMA control charts	We Included a table summarizing the key findings from the comparative analysis of EWMA control charts
Ensure that the mathematical notation is consistent throughout the manuscript	We made the mathematical notations consistent throughout the manuscript.
Clarify the role of the smoothing constant (λ) in the EWMA statistics.	We clarified the role of the smoothing constant (λ) in the EWMA statistics.
Discuss how the proposed method can be	We made separate heading of the

adapted for different types of data distributions.	proposed method can be adapted for different types of data distributions.
Include a section on future research directions based on the findings of this study.	We made heading of future research directions based on the findings of this study.
Ensure that the abstract provides a concise summary of the methodology and key results	We changed almost completely abstract. Now it show a concise summary of the methodology and key results.
Revisit the conclusion to emphasize the practical applications of the proposed control charts.	We rewrote the conclusion to emphasize the practical applications of the proposed control charts.
Provide a detailed description of the software and tools used for the simulation study.	We added detailed description of the software and various tools used for the simulation study.
Ensure that all references are cited correctly and are relevant to the study.	We checked again references and tried to write it correctly.
Review the manuscript for grammatical errors and improve the overall readability.	We review the manuscript for grammatical errors and improve the overall readability.
